# Utilization of beef lung powder in model chicken emulsion formulation

**DOI:** 10.1002/fsn3.4478

**Published:** 2024-09-18

**Authors:** Cem Okan Özer, Ganime Beyzanur Var, Kamil Emre Gerçekaslan, Ezgi Demir Özer

**Affiliations:** ^1^ Department of Food Engineering, Faculty of Engineering and Architecture Nevsehir Hacı Bektaş Veli University Nevsehir Turkey; ^2^ Department of Gastronomy and Culinary Arts, School of Applied Science Cappadocia University Nevsehir Turkey

**Keywords:** emulsified meat products, meat by‐products, protein functionality

## Abstract

The present study aimed to demonstrate the effect of beef lung powder addition to model chicken emulsion formulations on quality parameters and determine the optimum usage dose. A mixture design method was employed to determine the ideal proportions of chicken meat, animal fat, and water. The optimal formulation comprises 70.48% chicken meat, 12.42% animal fat, 9.30% water/ice, and 7.80% beef lung powder (w/w). The predicted outcomes for this optimized emulsion include a cooking loss of 3.05%, emulsion stability of 85.21%, an oxidation rate increase of 2.93%, a color difference of 13.24%, and firmness of 24.16 N. The use of lung powder resulted in a reduction in cooking loss and an increase in emulsion stability and hardness. Nevertheless, an increase in both color change and oxidation rate was found in emulsion models. The results of this study demonstrate that beef lung powder is a highly functional ingredient with the capacity to significantly enhance the stability and texture of chicken emulsions. Furthermore, it has the potential to considerably improve the nutritional and quality attributes of emulsified meat products, therefore suggesting its broader applicability in food production.

## Graphical abstract

This study examines the effects of adding beef lung powder to chicken emulsion formulations, revealing significant improvements in emulsion stability, reduced oxidation rates, and minimized cooking losses. The optimal formulation includes 70.48% chicken meat, 12.42% animal fat, 9.30% water/ice, and 7.80% beef lung powder, demonstrating that beef lung powder can enhance both the physical and nutritional qualities of emulsified meat products.

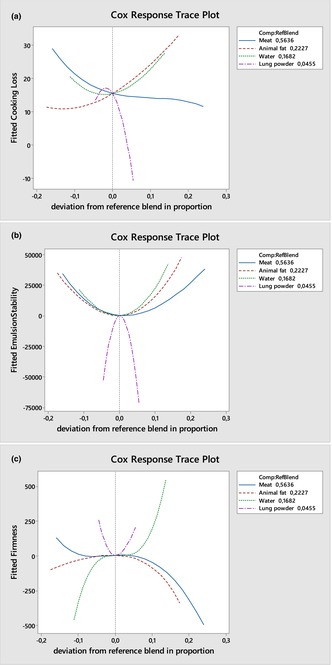

## 1 INTRODUCTION

Meat production and consumption have been rising steadily due to the growth of the world population. It was reported that the production of meat has increased to three times the levels in the last 60 years. In 2021, the global production of meat reached 350 million tons, with beef accounting for approximately 20% of this total. It is anticipated that by 2030, meat production will be 14% higher than the current level (FAO, 2022; OECD, 2021; Ritchie et al., 2017). Approximately 50% of cattle's live weight is considered suitable for human consumption, while the remaining 50%, referred to as waste and by‐products, is not intended for direct human consumption (Albertí et al., 2008). The wastes and by‐products resulting from slaughterhouse processing include blood, bones, fat, some organs, skin, stomach and intestines. These wastes and by‐products are a type of protein‐ and lipid‐rich organic waste and are mostly utilized to produce value‐added by‐products such as fertilizer or animal feed (Salminen & Rintala, 2002; Wang et al., 2022). However, it is essential to reduce the amount of these valuable waste and by‐products and to increase their potential use as human food to ensure the sustainability of food production and consumption and to protect the environment (Al‐Zohairi et al., 2023). This can contribute to ensuring environmental, social and economic sustainability in animal production (Kumar et al., 2023).

Beef lung is a significant by‐product of the slaughterhouse with high protein content consisting of essential amino acids, high biologically available iron and some critical minerals such as potassium, phosphorus, sulfur and sodium (Cardoso‐Santiago & Arêas, 2001; Jayawardena, 2020). Nevertheless, the dietary availability and consumption of beef lungs are quite limited due to concerns about pathogens and contaminants that may be present in lung tissue. Therefore, it is of great importance for sustainable meat production to recycle this valuable meat by‐product safely, with minimal environmental impact and economic cost. (Toldrá et al., 2021). Researchers stated that beef lung, with its high processing potential and nutritional value, could be utilized as an ingredient in food products to overcome health problems such as malnutrition or anemia (Darine et al., 2010; Moreira‐Araújo et al., 2008). Jayawardena et al. (2022) indicated that a potential food ingredient with high water‐binding capacity, protein, and heme‐iron content could be produced from beef lungs. Moreover, it has been evidenced that this ingredient could improve the functional and nutritional quality of pasta (Jayawardena et al., 2019). On the other hand, Darine et al. (2010) suggest that lung proteins have the capacity to enhance some quality parameters in meat products, particularly emulsification. Nevertheless, it has been argued that the potential of the lung is still not sufficiently utilized and therefore does not currently exist in food technology as a valuable by‐product (Jayawardena et al., 2022). At this stage, it can be concluded that there is a lack of research on the use of the lung in meat products. Although studies have been conducted to determine some properties of proteins derived from lung (Arêas et al., 1993; Darine et al., 2010), the effect of using lung in a meat product on product quality has not been investigated. It is thought that lung, with its high protein content, may be an important component in improving the quality of emulsified meat products. Protein is essential for enhancing and maintaining the stability of meat emulsions due to its amphiphilic and film‐forming abilities, as well as its interfacial adsorption properties (Zhang, Zhao, et al., 2022). Therefore, it is a key ingredient in the formation and stabilization of meat emulsions (Lam & Nickerson, 2013; Liu et al., 2011). The properties of lung powder, including protein content, water holding capacity and water binding capacity, which significantly influence emulsion characteristics, suggest that it may have potential as a substitute or main component in meat emulsions and to improve emulsion properties.

This study aimed to improve the emulsion ability of chicken trimmed meats by adding beef lung powder. For this purpose, the potential of using lung powder in the emulsion model together with chicken trimmed meats, which are considered as low‐quality meats, was investigated. Moreover, the recovery of beef lungs, which are not at the desired level for use as human food but have high nutritional content, and increasing the nutritional value of food are other aims of the study. Therefore, the investigation has been designed to evaluate the impact of beef lung powder on a variety of parameters, including emulsion stability, cooking loss, color differences, oxidation rate and hardness values. Additionally, the objective is to determine the optimal beef lung powder ratio for achieving the desired results.

## 2 MATERIALS AND METHODS

### 2.1 Materials

The beef lung and fat were provided by a commercial facility (Erdem Et) that utilizes hygienic slaughtering techniques. The lungs and back fat used in the study were obtained from healthy male cattle carcasses at 24 h post‐mortem. Furthermore, 24 h post‐mortem and Ross breaded broiler chicken carcasses, slaughtered by standard industrial procedures, were obtained from a local meat processor (Eroğlu Tavukculuk). The trimmed meat obtained during the dissection of the carcass to obtain the primary cuts such as breast, tenderloin, back, wing, leg and drumstick was used in the production of model chicken emulsions.

### 2.2 Production of beef lung powder

Firstly, the beef lung was washed several times with distilled water and citric acid solution (% 0.2, w/w) in order to remove the blood and other tissue fragments and reduce microbial risks (Zaki et al., 2015). After removing the tracheas and excess tissues, the lungs were cut into smaller cubic pieces (approx. 2 cm^3^). Lung pieces were dried with a lyophilizer at −80°C and 0.01 mbar pressure (Operon, OPR‐FDU‐8612, Korea). The drying process continued until the moisture content fell below 10%. The freeze‐dried lungs were ground using a Waring Blender (Waring 8011 EB Blender) at 18000 rpm for 30 s to pass through a 100‐mesh sieve. The powder was stored with vacuum‐sealed at 4°C.

### 2.3 Production of model chicken emulsion system

The model chicken emulsion system was produced as described by Cofrades et al. (2008) with some changes. The preparation of the model chicken emulsion was carried out as follows: the chilled chicken trimmed meat was ground in a Waring blender (Waring Lab. Sci.) at 18,000 rpm for 1.5 min. Next, sodium chloride was added to the meat and mixed for another 30 s. Then, chilled beef fat was added to the mixture and homogenized for 1.5 min. Subsequently, ice was added, and the emulsion mixture was homogenized for 2 min. In the formulation of the model chicken emulsion, trimmed chicken meat, beef fat, water, and beef lung powder were used by the experimental design (Table 1), with the specified proportions. Sodium chloride at a dose of 1.5% (w/w) of the total emulsion weight was used in each trial. In each experiment, chicken trimmed meat, fat and blender jar were used that were strictly chilled to 0–4°C to prevent excessive temperature increase during emulsion production. Additionally, the emulsion temperature was also continuously monitored with a digital thermometer to confirm that the temperature of the emulsions never exceeded 12°C. The obtained emulsion mixture was transferred into plastic Falcon centrifuge tubes in portions of 25 ± 1 grams and centrifuged at 4500 rpm for 15 min (Hanil Combi 514R). Thereafter, the samples were heat treated in a hot water bath (JSR) for 30 min until the core temperature reached 72°C. Finally, the samples were rapidly cooled and stored with vacuum‐sealed at 4°C for 7 days.

**TABLE 1:** Experimental design for the production of chicken meat emulsion systems and experimental data for the responses.

Standard order	Factor 1 (X_1_) chicken meat (%)	Factor 2 (X_2_) animal fat (%)	Factor 3 (X_3_) water (%)	Factor 4 (X_4_) beef lung powder (%)	Response I (Y_1_) color differences* (ΔE)	Response II (Y_2_) cooking loss* (%)	Response III (Y_3_) emulsion stability*	Response IV (Y_4_) firmness* (N)	Response V (Y_5_) oxidation rate* (%)
1	48.18	26.14	23.41	2.27	8.58	27.50	67.54	20.32	4.90
2	40.00	40.00	20.00	0.00	0.94	18.90	66.70	13.43	5.26
3	55.00	40.00	5.00	0.00	0.61	29.38	72.47	18.26	5.09
4	40.00	40.00	10.00	10.00	29.05	20.70	74.44	14.46	3.57
5	48.18	21.14	23.41	7.27	22.95	15.00	73.40	17.02	6.11
6	48.18	31.14	18.41	2.27	11.93	21.25	69.59	12.79	5.45
7	48.18	31.14	13.41	7.27	21.37	18.00	74.25	14.82	3.97
8	60.68	13.64	23.41	2.27	10.13	15.50	72.61	14.09	4.02
9	40.00	20.00	30.00	10.00	27.13	28.25	68.77	15.58	4.46
10	68.18	18.64	10.91	2.27	15.62	20.75	78.31	18.14	4.08
11	56.36	22.27	16.82	4.55	15.48	11.00	73.77	17.98	4.60
12	55.00	5.00	30.00	10.00	25.93	9.38	82.05	19.29	5.04
13	80.00	15.00	5.00	0.00	2.16	4.38	82.37	22.96	3.60
14	80.00	5.00	5.00	10.00	26.58	6.84	91.83	37.79	4.93
15	80.00	5.00	15.00	0.00	1.65	6.50	79.72	16.53	3.47
16	55.68	31.14	10.91	2.27	10.51	17.52	70.08	15.51	4.27
17	55.68	13.64	23.41	7.27	22.00	10.37	75.77	17.10	5.61
18	40.00	30.00	30.00	0.00	1.24	7.25	63.92	6.66	3.91
19	45.00	40.00	5.00	10.00	31.98	20.47	75.40	25.80	5.47
20	65.00	5.00	30.00	0.00	1.90	19.98	66.36	6.13	4.66
21	68.18	13.64	10.91	7.27	25.49	2.75	83.30	27.48	4.88
22	68.18	13.64	15.91	2.27	12.32	11.25	74.21	18.33	4.34
23	50.68	31.14	10.91	7.27	23.44	17.25	75.05	16.12	4.94

*The values presented are the mean values obtained from two replications.

### 2.4 Physicochemical analysis

The protein, moisture, ash and fat content for beef lung powder and model chicken emulsion were determined by the official AOAC methods (AOAC, 2010). The pH of the samples was determined by a digital pH meter (PL‐700 PV, Taiwan) by mixing the 10 g sample in 20 mL of distilled water. The color properties (L*, a*, b*) of beef lung powder and model emulsion system were measured by a Hunter colorimeter (CR400, Konica Minolta). The color differences (ΔE) of the samples compared to model chicken emulsion without beef lung powder were calculated with the following formula:

$$∆E=\sqrt{{\left({∆}_{L*}\right)}^{2}+{\left({∆}_{a*}\right)}^{2}+{\left({∆}_{b*}\right)}^{2}}$$

Additionally, the heme and total iron content (μg/g sample) of the lung powder samples were determined (Hornsey, 1956). Briefly, 20 mL of an acidified acetone solution was added into 10 grams of the sample and vortexed for 1 min. The mixture was then incubated in the dark for 1 hour, after which the resulting extract was subjected to centrifugation at 2200 rpm for 15 min. Subsequently, filtration was conducted through Whatman No. 3 filter paper, and the absorbance was determined at 640 nm. The absorbance value was multiplied by a factor of 6800 and divided by the sample weight to calculate the concentration of total pigments (μg haematin/g sample). A factor of 0.0882 was used to calculate iron content (μg iron per μg hematin).

### 2.5 Water solubility and binding of beef lung powder

The water solubility index (WSI) and water binding capacity (WBC) of bovine lung powder were determined with minor modifications to the methods described by Jayawardena et al. (2022) and Mahdavi et al. (2016), respectively. Firstly, a mixture of 5 g of beef lung powder in 20 mL of distilled water was prepared and centrifuged (Hanil Combi 514R) at 8000 rpm for 30 min. After the centrifugation, the supernatant and pellet were separated. The weight of the pellet was determined in order to calculate the WBC. And the weight of the supernatant was determined after drying at 105°C in order to calculate the WSI. WSI and WBC of beef lung powder were determined using the following formulas;

$$\mathrm{WSI}=\left({\mathrm{\Delta W}}_{\text{supernatant weight difference}}/W_{\text{sample}}\right)*100$$

$$\mathrm{WBC}=(\left(W_{\text{sample}}–W_{\text{pellet}}/W_{\text{sample}}\right)*100$$

### 2.6 Emulsion stability and cooking loss

Emulsion stability (ES) and cooking loss for the model emulsion system were determined as described by Colmenero et al. (2005). 25 g of raw emulsion mixture was weighed into centrifuge tubes and centrifuged (Hanil Combi 514R) at 3000 rpm for 5 min. Then, the mixture was heat treated until the core temperature reached 72°C. After the heat treatment, all the exudate in the tube was separated and weighed. After that, the samples were heated at 100°C for 12 h, and the amount of liquid separated was determined as fat exudate. The water released was calculated as the difference between the total liquid exudate and the fat exudate. Exudate water and fat were expressed as % of the sample weight. Emulsion stability was calculated as follows;

$$\mathrm{ES}\;\left(\%\right)=100–\left(\text{Total of water and}\;\mathrm{fat}\;\text{released}\right)$$

The cooking loss of the model emulsion system was determined by calculating the proportion of weight loss during heat treatment to the initial weight.

### 2.7 Lipid oxidation

The lipid oxidation levels of the model emulsion system samples were evaluated by detecting thiobarbituric acid reactive substances (TBARS) using the method described by Kilic and Richards (2003). In brief, 2 g of the sample was homogenized with 12 mL of a trichloroacetic acid solution for 30 s and then was filtered using Whatman No. 1 filter paper. 3 mL of the filtrate and 3 mL of the thiobarbituric acid solution were mixed and incubated at 100°C for 40 min. The mixture was cooled and centrifuged at 2000 rpm for 5 min. The absorbances of the samples were then determined at 530 nm. The results are reported in μmol malondialdehyde (MDA)/kg of sample. The study determined the increase in oxidation level of the experimental groups based on the oxidation level of the emulsion model system without lung powder. The increase rate determined in the oxidation level of the experimental groups was evaluated as a response in the response surface method.

### 2.8 Texture profile analysis

A Warner‐Bratzler shear force analysis was conducted on model chicken emulsion samples using the TA‐XT.plus Texture Analyzer (Stable Micro Systems Ltd), which was equipped with a Warner‐Bratzler knife set (HDP/WBV). Samples were prepared by cutting a length of 30 mm and a diameter of 17 mm. The cutting force (firmness, N) and work of shear (N.s) values were calculated from the graph obtained under the test conditions: test speed 1.5 mm/s, cutting distance 30 mm and trigger force 30 g. The firmness values (cutting force) were evaluated as a response in the response surface method.

### 2.9 Microbiological analysis

Total mesophilic aerobic bacteria, coliform bacteria and yeast and mold count were determined for fresh beef lung and dry beef lung powder. 10 grams of the sample were homogenized with 90 mL of peptone water for 3 min. Then, a tenfold dilution series was prepared from the homogenate, and suitable dilutions were inoculated using the spread plate technique. The total mesophilic aerobic bacteria, coliform bacteria, and total yeast‐mold counts were counted using plate count agar at 30°C for 48 h, violet red bile agar at 37°C for 24 h, and potato dextrose agar at 25°C for 72 h, respectively. Results are expressed as colony‐forming units per gram of sample (log CFU/g sample).

### 2.10 Statistical analysis

The objective of the present study was to investigate the potential of using lung powder in combination with chicken meat, animal fat, and water in the chicken emulsion model formulation. The effects of the ratios of chicken trimmed meat (X_1_), animal fat (X_2_), water (X_3_), and beef lung powder (X_4_) in the emulsion formulation on emulsion stability, cooking loss, color differences, oxidation rate, and hardness values were investigated, and the optimum lung powder ratio was determined. In order to carry out this investigation, the extreme vertices mixture design (Minitab 17, Minitab LLC) was used. The experimental design consisted 23 experimental groups for 4 factors at varying levels, as shown in Table 1. In the study, the mean of at least three repetitions for each response was presented as the result. The experiment results were analyzed and fitted to a regression model that included coefficients for linear, quadratic and special cubic effects. The best‐fitting models were determined by mixture regression. The regression model for the response variable (*Z*) of the four independent variables can be represented by the following polynomial equation:

(1) $$Z=\sum_{i=1}^{4}\beta_{i}X_{i}+\sum_{i=1}^{4}\beta_{\mathrm{ii}}X_{i}^{2}+\sum_{i=1}^{4}\beta_{\mathrm{iii}}X_{i}^{3}$$

*Z* represents the predicted responses, including color differences, cooking loss, emulsion stability, firmness, and oxidation rate. The linear, quadratic and the cubic coefficients were presented by *β*_*i*_, *β*_*ii*_ and *β*_*iii*_, respectively. The model and fitness of the model were tested using analysis of variance (ANOVA) test and coefficients (*R* ^2^, adjusted *R* ^2^ and estimated *R* ^2^). Additionally, the *p* and *F*‐values (probability and Fisher test value) were considered determine the adequacy and significance of the regression model and to obtain a statistically significant regression model.

## 3 RESULTS AND DISCUSSION

The chemical composition, physicochemical characteristics and color values of the lung powder used in this study were determined. The moisture value reached at the end of drying and the drying technique applied directly affect the proximate composition and characteristics of the lung powder. The present results are similar when comparing the moisture (1.83 ± 0.18%) and ash content (5.29 ± 0.13%) obtained with those of the lung powder obtained by conventional drying by Jayawardena et al. (2022). Additionally, compared to the conventionally dried method, lyophilized dried beef lung powder was found to have relatively higher L* (64.82 ± 0.51) and a* (11.63 ± 0.46) values and lower TBARS (1.34 ± 0.21 μmol MDA/kg powder) values (Jayawardena, 2020). The high protein (82.34 ± 0.79%), haem iron (217.06 ± 2.06 μg/g) and total iron (651.19 ± 6.19 μg/g) content of lung powder indicate that it has a significant potential to increase the nutritional content of many food products. Furthermore, high water binding capacity (294.27 ± 2.68%) and water solubility index (71.89 ± 0.03%) provide additional evidence to support the hypothesis that it can be utilized to improve the quality parameters of emulsion systems.

The results of the microbiological analysis for the fresh beef lung showed the presence of 5.49, 2.21, and 3.19 log CFU/g samples of total mesophilic aerobic bacteria, coliform bacteria, and total yeast‐mold, respectively. However, the microbial load of the dried beef lung was found to be at a lower level than that of the fresh lung. It was determined that the beef lung powder had 2.01, 1.18 and 1.66 log CFU/g samples of total mesophilic aerobic bacteria, coliform bacteria, and total yeast‐mold, respectively. It is thought to be due to the effect of the washing process using a citric acid solution and the freezing process during the drying process. It was reported that citric acid applications are effective on many bacteria such as *Escherichia coli*, *Salmonella* and *Staphylococcus* count in different food products (Al‐Rousan et al., 2018; Mani‐López et al., 2012; Olaimat et al., 2017). In addition, the destruction effect of the lyophilization process on microorganisms has also been reported (Tejada et al., 2008).

Experimental groups, factors and responses were shown in Table 1. The components of the mixture were used as model terms and the mixture regression was used as the model fitting. Linear, quadratic and special cubic models and interaction effects of the independent variables were fitted to evaluate the ratio of beef, animal fat, water, and beef lung powder. The high levels of *R* ^2^, adjusted *R* ^2^ and predicted *R* ^2^ values obtained from the models, which are important indicators for the fit of the models, indicate that models are highly fitted. Furthermore, the obtained *p* and *F* values from ANOVA also indicated the adequacy and significance of the regression model and its coefficients (<0.05) (Table 2).

**TABLE 2:** Regression coefficients of predicted models for the investigated responses of chicken meat emulsion system with lung powder and independent effects of the factors.

Variables^a^	Coefficient for color differences (ΔE)	Coefficient for cooking loss	Coefficient for emulsion stability	Coefficient for firmness	Coefficient for oxidation rate
Linear					
β_1_	−77	−8	107	17	495
*β*_2_	−451	168	116	−27	2696
*β*_3_	−707	97	108	−95	4462
*β*_4_	10,573	−8197	−893	10,928	−75,988
Quadratic					
*β*_12_	900*	−124	−142*	–	−5302*
*β*_13_	1211*	–	−174*	–	−7506*
*β*_14_	−11099*	9662*	1017*	−11966*	65444*
*β*_23_	1402*	−571	–	–	−6695*
*β*_24_	−10955*	10894*	1259*	−13940*	94554*
*β*_34_	−11168*	16411*	–	−11811*	94163*
Special Cubic					
*β*_123_	–	–	–	4810*	−5607*
*β*_124_	–	−5599	−339	4517*	–
*β*_134_	–	−14823*	–	–	–
*β*_234_	–	−6762	–	–	−31878*
*R* ^2^	.99	.87	.98	.91	.85
Pre‐ *R* ^2^	.97	.52	.95	.59	.56
Adj‐*R* ^2^	.98	.73	.97	.84	.64
*F* value	130.18	6.48	71.80	13.90	3.99
*p* value	.001	.002	.001	.001	.002

Abbreviations: *β*_1_: chicken meat; *β*_2_, animal fat; *β*_3_, water/ice; *β*_4_, lung powder; *β*_12_, interaction for chicken meat and animal fat; *β*_13_, interaction for chicken meat and water/ice; *β*_14_, interaction for chicken meat and lung powder; *β*_23_, interaction for animal fat and water/ice; *β*_24_, interaction for animal fat and lung powder; *β*_34_, interaction for water/ice and lung powder; *β*_123_, interaction for chicken meat, animal fat and water/ice; *β*_124_, interaction for chicken meat, animal fat and lung powder; *β*_134_, interaction for chicken meat, water/ice and lung powder; *β*_234_, interaction for animal fat, water/ice and lung powder.

^a^ Polynomial model adjusted by mixture regression at the level of 0.1% with the lack‐of‐fit test, where, *β*_i_ is the linear coefficient (main effect), *β*_ij_ is the two factors interaction coefficient and *β*_ijk_ is the three factors interaction coefficient.

*Significant at *p* ≤ .05.

Regression models obtained using mixture design frequently include linear, quadratic, and special cubic forms. The objective is to develop models that provide a more comprehensive analysis of the significant factors involved. This design primarily provides the opportunity to measure the first‐order coefficients in relation to the factors. Moreover, the quadratic and special cubic models allow for the estimation of linear and all two‐interaction terms, as well as linear, two‐ and three‐interaction terms, respectively. This approach allows for the determination of both the pure effect of the factors and as well as the assessment of any potential synergistic or antagonistic interactions (Oehlert, 2010; Pagés‐Díaz et al., 2014).

**FIGURE 1:**
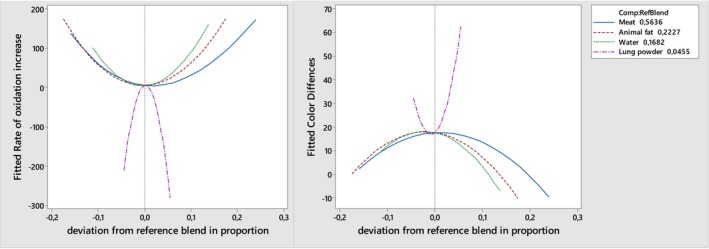
Response trace plots for the effect on rate of oxidation increase and color differences of emulsion formulations.

The analysis results of the regression model showed that positive and negative quadratic and specific cubic interactions of all ingredients in the emulsion formulation were effective on the responses (*p* < .05), but their linear effects were not significant (Table 2). It is noteworthy that all quadratic effects for all factors that affect the color differences of the emulsion system exhibit an opposite trend with the oxidation rate (*p* < .05). Moreover, all interaction effects containing beef lung powder had a negative effect on the color differences values, while they had a positive effect on the oxidation rate. The correlation between the changes in color and oxidation caused by the use of lung powder in the emulsion model system is better seen in the trace plot (Figure 1). It seems that the change in color differences of the emulsion model system is not only dependent on the color characteristics of the lung powder used but also on increasing oxidation rate of emulsion. Even minor changes in the amount of lung powder in the formulation can cause significant changes in the color and oxidation values of the emulsion. The regression model (Table 2) and trace plot (Figure 1) show that these changes can be positive or negative due to the effect of quadratic and cubic interactions. This indicates that while an increase in the amount of lung powder results in an increase in color difference and a decrease in oxidation up to a certain level, after this level is reached, the situation changes and the opposite occurs. Nevertheless, an analysis of the oxidation rate responses indicated that the cubic interactions between the oil, water and lung powder had a strong impact on the model, in addition to the quadratic effects. This may be related to the color properties and iron content of the lung powder used. The lung powder used had lower L* and a* values and relatively higher b* values compared to the emulsion model produced. The transformation of myoglobin into metmyoglobin during drying process may be a primary cause for the change in the color properties of lung powder. Therefore, its inclusion in the formulation, especially in high proportions, leads to significant color differences in the model emulsion system. Similarly, Jayawardena et al. (2019) also reported that L* and b* values decreased and a* value increased by using lung powder in pasta formulation. As a result, visible color differences were reported in pasta produced with lung powder. On the other hand, given that color changes are typically correlated with oxidation processes, the relationship between color differences and oxidation rate in presented results can also be regarded as an indirect relationship (Tushar et al., 2023). In addition to all of these, beef lung powder is highly susceptible to oxidation due to its chemical composition (Jayawardena et al., 2022). It is widely acknowledged that iron, particularly in its heme form, has the capacity to catalyze lipid peroxidation through various mechanisms, including Fenton‐like and iron(III)/iron(IV) reactions (Xiong et al., 2020). Similarly, Jayawardena et al. (2022) indicated that dried lung powder had low lipid and protein oxidative stability and TBARS levels significantly increased during storage. However, it is also known that proteins in emulsions have an antioxidant effect (Feng et al., 2021). In emulsion systems, proteins are closer to reactive oxygen species or free radicals than unsaturated fatty acids. Therefore, proteins are considerably more susceptible to react with them or bind to metal ions. This makes proteins in emulsified products more susceptible to oxidation and thus could prevent or delay lipid oxidation by oxidizing before unsaturated fatty acids (Genot et al., 2013; Gülseren & Corredig, 2014; Zhang, Wang, et al., 2022). In addition, the antioxidant effect of the proteins forming the physical layer on the fat globules in the emulsions and their role in preventing oxidation are also very important (Mackie et al., 1991). The thickness of this physical layer increases with increasing protein concentration, resulting in increased antioxidant activity (Schröder et al., 2017). On the other hand, an excessive increase in protein content may also serve to enhance the catalyzing ability of iron in the process of lipid oxidation. The release of iron from high‐molecular‐weight proteins at the point of binding to the protein surface and the lecitic phosphoric region has been found to promote the oxidation of lipids (Xiong et al., 2020). Consequently, it is essential to include cubic effects in the model in order to accurately describe the complex dynamics of the oxidation rate in response to changes in multiple variables and their interactions. It is therefore possible to consider the potential negative effects on color and oxidation stability of high protein lung powder used in meat products as opportunities for improvement by determining the optimum dosage.

**FIGURE 2:**
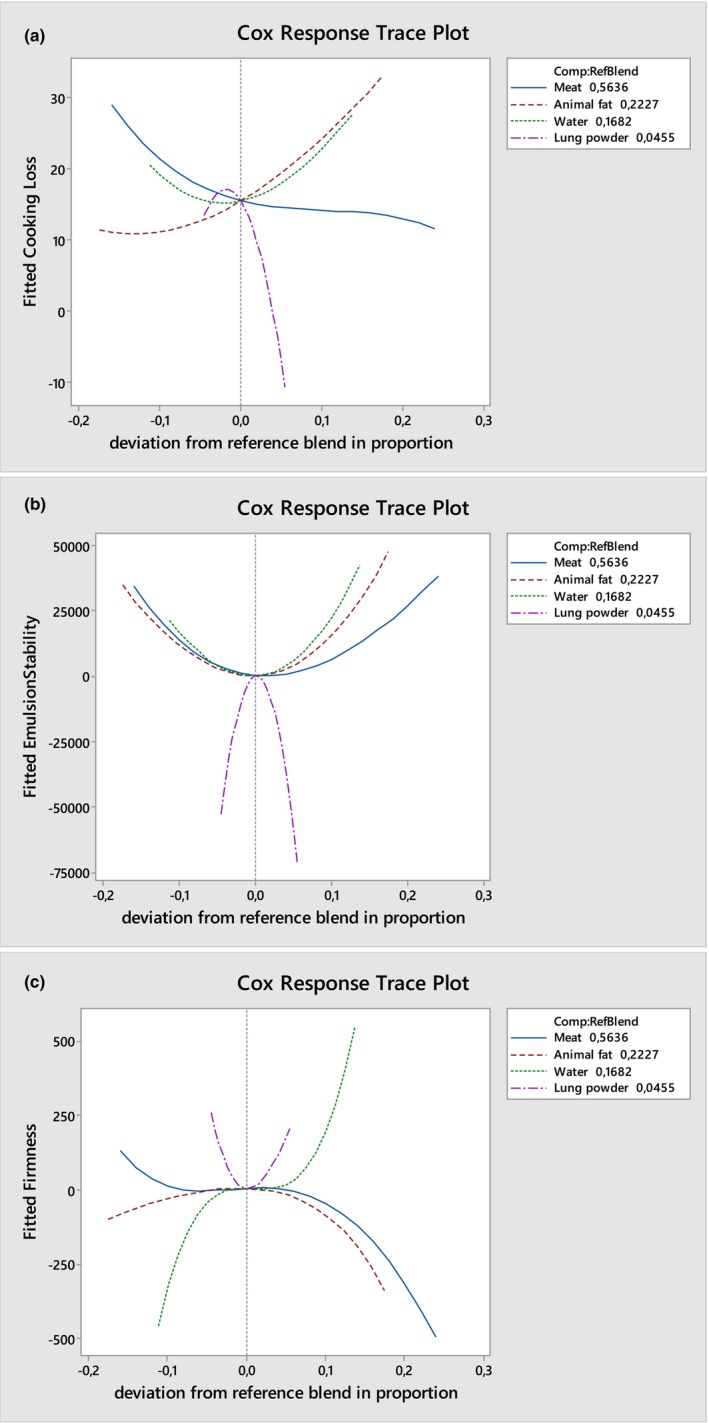
Response trace plots for the effect on rate of cooking loss (a), emulsion stability (b) and firmness (c) of emulsion formulations.

The results showed that all quadratic interactions involving lung powder have significant effects on cooking loss and firmness values (*p* < .05) (Table 2). In addition, the special cubic interactions between the amount of chicken trimmed meat and other ingredients in the emulsion had significant effects on the firmness and cooking loss value of the emulsion (*p* < .05). A notable aspect of the findings is the observation that the interactions identified as significantly influencing cooking loss and firmness exhibit opposing characteristics. In summary, the interactions affecting cooking loss are positive, whereas those influencing firmness are negative (Figure 2). Some studies have indicated that as cooking loss decreases, the hardness of the emulsion increases. This suggests that there may be an inverse relationship between cooking loss and firmness. It is thought that a stable emulsion that retains moisture can maintain its structural stability during the cooking process, which results in a harder product (Álvarez et al., 2007; Kim et al., 2018).

It is also noteworthy that the cubic interaction between the meat, water and beef lung powder ratio had a considerable impact on the cooking loss of the emulsion (*p* < .05) while the cubic effect of all factors on firmness is relatively insignificant. These findings indicated that alterations in the fat ratio within the emulsion formulation, through the incorporation of beef lung powder, may result in a slight reduction in cooking loss and indirectly in firmness values (Figure 2). However, when low‐fat emulsions are cooked, the stability of the emulsions usually decreases due to the decrease in fat and water, which negatively affects the quality of the final product (Álvarez et al., 2007; Álvarez & Barbut, 2013). In meat emulsions, the protein is transformed into a gel‐like structure during the cooking process, which helps to retain moisture. As collagen denatures and gelatinizes, it forms a network that traps water, thereby reducing the loss of moisture during thermal processing (Ahmad et al., 2023; Ren et al., 2022). Therefore, it is thought that beef lung powder improves cooking loss and hardness with its high protein and especially collagen content and water retention capacity.

The results showed that linear and cubic effects for all factors had no significant effect on emulsion stability. However, some quadratic interactions have significant effects on emulsion stability (*p* < .05) (Table 2). When all interactions included in the present study are considered, it can be stated that second‐order interactions, which are of importance with regard to emulsion stability, have relatively small coefficients and therefore are less effective. In addition, it found that interactions of lung powder with chicken trimmed meat and fat have higher positive coefficients (Figure 2). Lung powder is considered to enhance emulsion stability due to its high protein content and water binding capacity. It is also thought that the proteins in lung powder, with their excellent emulsifying properties, contribute to increasing emulsion stability. Darine et al. (2010) stated that lung proteins contain a low molecular weight fraction and have high solubility and strong hydrophobicity with low surface and interfacial tensions. In additon, collagen is a significant part, accounting for more than 20% of lung proteins (Darine et al., 2010). And, in the present study, it is thought that the use of lung powder improved the emulsion stability due to its collagen content. Collagen has poor emulsifying properties, but it can be a significant alternative emulsifier (Kim & Park, 2004). Additionally, it is known that collagen cross‐links covalently in aqueous solutions, forming swellable matrices and thus forming hydrogel structures (Gómez‐Guillén et al., 2011). Thus, it reduces the surface tension between liquid and air and increases the viscosity of the aqueous phase. These properties, combined with its ability to bind water, can contribute to the stabilization of emulsified products (Gómez‐Guillén et al., 2011). Furthermore, the degree of cross‐linking within the collagen structure is directly correlated with its capacity to bind water. An increase in the degree of cross‐linking within the collagen structure has been observed to result in a reduction in the water retention capacity of the collagen, leading to a decline in the elasticity and texture of the emulsion (Schroepfer & Meyer, 2017). This may be the reason the interaction of water and lung powder exhibits a negative effect on firmness of emulsions (Table 2).

One of the important results of the present study was that among all the factors determined to have a significant effect on the responses, the regression coefficient, therefore the factors with the greatest effect on the response, were the interactions including lung powder (*p* < .05). Furthermore, the direction of all interactions, positive or negative, including lung powder on the responses was consistently the same trend. Although not statistically significant, the finding that the linear effect of lung powder exhibited higher regression coefficients than other components significant, is also consistent with this result. However, the quadratic interactions between lung powder and the variables of meat and fat content had a considerable impact on all the response variables tested (Table 2). The effects exhibited relatively large coefficients and were either positive or negative, depending on the response. This indicates that the influence of lung powder on the formulation is both multifactorial and of considerable consequence. Accordingly, it is critical to optimize the amount of use in order to effectively assess the potential impact of lung dust on the emulsion. The highest firmness and emulsion stability and the lowest oxidation rate, cooking loss and color differences were preferred to obtain the optimum model chicken emulsion formulation with lung powder. Multiple response optimizers for the identified parameters were used to derive the optimal values for all responses. The optimization process was conducted in the Minitab statistical software. The optimal formulation was determined to be 70.48% chicken trimmed meat, 12.42% animal fat, 9.30% water/ice and 7.80% beef lung powder. The model predicted that the chicken emulsion produced with optimum formulation will have a cooking loss of 3.05%, emulsion stability of 85.21%, oxidation rate of 2.93%, color difference of 13.24, and firmness values of 24.16 N. The presented *R* ^2^ values suggested high accuracy in predicting all responses, particularly for color differences and emulsion stability.

## 4 CONCLUSION

The results of the study demonstrated that the ingredients in the model chicken emulsion have some synergistic and antagonistic effects on the emulsion properties. It is hypothesized that the use of the optimal formulation could result in the production of a model chicken emulsion with high firmness, emulsion and oxidation stability, low cooking loss and color changes. The results of the study indicate that the utilization of beef lung powder in model chicken emulsion not only provides a sustainable method for the utilization of low‐value meat by‐products but also represents a valuable addition to emulsified meat products, enhancing their functional properties and nutritional content. This could represent a significant alternative in producing healthier, more sustainable meat products, meeting consumer and industry demands for quality, affordability, sustainable ingredients and reduced environmental impact of livestock products. In conclusion, in light of the increasing focus on research into sustainable food production systems, it is important to investigate the potential impact of bovine lung powder on the shelf life and consumer acceptance of the product, as well as its applicability in other food product formulations.

## AUTHOR CONTRIBUTIONS

Cem Okan Özer: Conceptualization, Investigation, Methodology, Performing experiments, Writing‐original draft, Writing–review & editing. Ganime Beyzanur Var: Methodology, Performing experiments, Writing–original draft, Writing–review & editing. Kamil Emre GerÇekaslan: Performing experiments, Writing–review & editing. Ezgi Demİr Özer: Conceptualization, Performing experiments, Writing–original draft, Writing–review & editing.

## FUNDING INFORMATION

The authors received no specific funding for this work.

## CONFLICT OF INTEREST STATEMENT

The authors declare that there is no conflict of interest.

## Data Availability

The data used in this study are presented in the article. Additional information and data are available from the corresponding author upon request.
